# Intrahepatic Fat Content and COVID-19 Lockdown in Adults with NAFLD and Metabolic Syndrome

**DOI:** 10.3390/nu14173462

**Published:** 2022-08-24

**Authors:** Sofía Montemayor, Catalina M. Mascaró, Lucía Ugarriza, Miguel Casares, Cristina Gómez, J. Alfredo Martínez, Josep A. Tur, Cristina Bouzas

**Affiliations:** 1 Research Group on Community Nutrition and Oxidative Stress, University of the Balearic Islands-IUNICS, 07122 Palma de Mallorca, Spain; 2 Health Institute of the Balearic Islands (IDISBA), 07120 Palma de Mallorca, Spain; 3 Camp Redó Primary Health Care Center, 07010 Palma de Mallorca, Spain; 4 Radiodiagnosis Service, Red Asistencial Juaneda, 07011 Palma de Mallorca, Spain; 5 Clinical Analysis Service, Universitary Hospital Son Espases, 07120 Palma de Mallorca, Spain; 6 Center for Nutrition Research, Department of Nutrition, Food Sciences, and Physiology, University of Navarra, 31008 Pamplona, Spain; 7 Cardiometabolics Precision Nutrition Program, IMDEA Food, CEI UAM-CSIC, 28049 Madrid, Spain; 8 CIBEROBN (Physiopathology of Obesity and Nutrition CB12/03/30038), Instituto de Salud Carlos III (ISCIII), 28029 Madrid, Spain

**Keywords:** COVID-19, lockdown, NAFLD, intrahepatic fat content, metabolic syndrome, dietary habits

## Abstract

Background: COVID-19 lockdowns had a significant impact on people’s health, triggering levels of anxiety, perceived stress, and changes in food and nutritional status. Objectives: To assess the changes in dietary habits, metabolic syndrome (MetS) and liver parameters before and after the COVID-19 lockdown according to changes in intrahepatic fat content in adults with non-alcoholic fatty liver disease (NAFLD) and MetS. Design: Pre- and post-lockdown observation of the COVID-19 lockdown on fifty-nine 40–60-year-old participants with MetS and NAFLD, in a parallel group, randomised experiment intended to treat NAFLD. Methods: Anthropometrics, liver and MetS biochemical parameters, intrahepatic fat content by abdominal magnetic resonance imaging, and dietary assessment using a validated 148-item Food Frequency Questionnaire were collected pre-COVID-19 lockdown and post-lockdown. Results: COVID-19 lockdown led to negative changes in the liver of patients with NAFLD and MetS, with weight gain and increases in glycemia, ALT and intrahepatic fat content post lockdown. Participants with worsened liver status had low consumption of fibre, cheese, nuts and coffee, and high consumption of sweets and pastries. Participants who improved liver status ameliorated ALT values, waist circumference, and intrahepatic fat content, assessed by magnetic resonance imaging post-lockdown. Conclusions: The maintenance of healthy lifestyle habits is vital, especially for populations with NAFLD and MetS, to reduce unhealthy lifestyle patterns displayed during lockdown.

## 1. Introduction

Non-alcoholic fatty liver disease (NAFLD) is the most common cause of chronic liver disease worldwide and causes triglycerides and free fatty acids to accumulate in the liver, increasing the risk of cardiovascular and liver-related death [1]. Its prevalence is 25.2% globally with wide geographical variation worldwide [2]. Its incidence has increased 5-fold lately, mainly among young adults [3], and it is increasing overall despite regional variation [4]. NAFLD affects the liver without excessive alcohol consumption, and ranges from simple fat accumulation (>5% of the hepatic parenchyma without injury to hepatocytes) to more advanced steatosis with associated fibrosis, cirrhosis, hepatitis, and hepatocellular cancer [5]. NAFLD is composed of non-alcoholic fatty liver and non-alcoholic steatohepatitis [5,6] and can be considered the hepatic manifestation of metabolic syndrome (MetS) [7]. The latest definition of MetS by the International Diabetes Federation (IDF) includes abdominal obesity defined by increased waist circumference (≥94 cm in men and ≥80 cm in women) and two or more of the following features: high blood pressure, fasting glucose or triglyceride concentrations, or low HDL cholesterol [8], and it increases the risk of cardiovascular disease, stroke, and type 2 diabetes [9].

Coronavirus disease 2019 (COVID-19) caused by severe acute respiratory syndrome coronavirus-2 (SARS-CoV-2) was declared a pandemic by the World Health Organization (WHO) in March 2020 [10]. This circumstance has prompted most governments throughout the world, including Spain, to adopt extraordinary measures, such as declaring a state of emergency and confining the whole population to their homes to control the spread of the disease [11]. These extraordinary measures such as the restriction of people’s social and personal lives had a major effect on their health. Numerous studies demonstrated that the SARS-CoV-2 disease lockdown caused levels of anxiety, depression, perceived stress, and dread to rise in the general population as well as larger changes in food and nutritional status [12,13]. The COVID-19 pandemic was able to change people’s eating behaviours and beliefs [14].

In Spain, the COVID-19 lockdown influenced food habits and lifestyles with potential negative health impacts [15]. During confinement, adherence to the Mediterranean diet increased, and consumption of homemade baking showed a higher increase, although consumption of ‘unhealthy’ food also increased, and the number of participants that practiced physical activity, as well as the time spent on it weekly, decreased [16]. However, the increased stress and anxiety generated by COVID-19 pandemics have also been linked to an increase in the consumption of alcohol and sweet foods, as well as an energy imbalance due to the lowered energy expenditure during lockdown [17]. Despite this, people who were usually less active increased physical activity in confinement and increased Mediterranean dietary patterns [15]. Then, although dietary habits improved during lockdown, unhealthy behaviours also increased [18].

Studies carried out in Italy found that during lockdown, restrictions on Italians’ lifestyles changed their psychological wellbeing [19]. Italians increased consumption of raw vegetables, whole grains, and water; however, there were also adverse trends, such as a high prevalence of sleeping difficulties [20], and increased consumption of processed “comfort foods”, such as chocolate, chips, and snacks [21,22]; sometimes this was due to anxiety about their eating habits during COVID-19 [23].

Therefore, given the lack of data in the literature on the impact of the lockdown on NAFLD and MetS patients, the aim of the current study was to assess the changes in dietary habits, metabolic syndrome, and liver parameters before and after the COVID-19 lockdown according to changes in intrahepatic fat content in adults with NAFLD and MetS.

## 2. Methods

### 2.1. Design

The present study is a pre- and post-lockdown observation of the COVID-19 lockdown, carried out in Mallorca (Balearic Islands, Spain) in a parallel group, randomised experiment intended to treat NAFLD. ClinicalTrials.gov (https://clinicaltrials.gov/ct2/show/NCT04442620 (accessed on 23 August 2022)) provides further protocol information [24], which was reviewed and approved by the Ethics Committee of the Balearic Islands (ref. IB 2251/14 PI) and followed the Declaration of Helsinki ethical standards. All participants were informed of the purpose and the implications of the study and provided written consent to participate.

### 2.2. Participants

The present analysis included 40–60-year-old men and women as those were who met at least three of the criteria for metabolic syndrome (MetS) as defined by the International Diabetes Federation Consensus Worldwide Definition of the Metabolic Syndrome [25], and had magnetic resonance imaging-confirmed NAFLD diagnoses (Signa Explorer 1.5T, General Electric Healthcare, Chicago, IL, USA). Prior cardiovascular disease, liver disease (other than NAFLD), cancer or a history of malignancy in the preceding five years, haemochromatosis, prior bariatric surgery, untreated depression, substance abuse, pregnancy, primary endocrinological diseases (other than untreated hypothyroidism), and severe psychiatric disorders (schizophrenia, bipolar disorder, eating disorders, or depression with hospitalisation within the preceding six months), concomitant therapy with steroids, or inability to provide informed consent were the exclusion criteria. In Mallorca (Balearic Islands, Spain), one-hundred and forty-three participants were contacted for an initial screening between June 2018 and January 2020, and fifty-nine of them qualified for the study. Due to their refusal to participate or failure to meet the inclusion criteria, 84 participants were omitted. The most recent data available prior to lockdown and the first measurements collected after lockdown were included in the analysis, provided they were taken within a four-month window prior to/after lockdown start/end.

### 2.3. Description of the COVID-19 Lockdown

On 14 March 2020, the Spanish government declared a state of alarm to manage the health crisis caused by COVID-19, and a home lockdown was imposed. People were advised to stay at home and work remotely due to severe limitations on movement. Almost everything was closed including schools, most companies, universities, gyms, and other facilities. The COVID-19 lockdown window was from 15 March to 4 May 2020. 

### 2.4. Dietary Assessment

Using a validated 148-item Food Frequency Questionnaire [26], data regarding intakes were gathered both before and during the lockdown. Participants were asked how frequently, on average, they consumed the amount of the item reported on the Food Frequency Questionnaire during the past year. They responded using nine options ranging from never or less than once per month to six or more times per day. The hundred and forty-eight items consist of typical portion sizes of foods and drinks with response categories to indicate frequency of consumption over a period of 12 months. Using a computer programme created by us based on readily available food composition data, energy and nutrient intakes were computed by multiplying the energy and nutrient composition of the portion size of each food item by the frequency of consumption [27]. 

### 2.5. Anthropometrics and Blood Pressure

Trained dietitians measured the participants’ weight, body mass index, waist circumference, blood pressure, and energy expenditure. At baseline, height was measured to the closest millimetre using a mobile stadiometer (Seca 213, SECA Deutschland, Hamburg, Germany), with the participant’s head held in the Frankfort plane. Participants wore light clothing and no shoes while having their weight evaluated using a Segmental Body Composition Analyzer for Impedance Testing (Tanita MC780P-MA, Tanita, Tokyo, Japan) (0.6 kg of weight was subtracted for their clothing). Participants were asked to stand upright while the waist circumference was measured halfway between the final rib and the iliac crest. BMI was determined as weight in kg/height in m^2^ (weight in kilogrammes divided by the square of height in meters). After a 5 min break in a seated position, blood pressure was assessed in triplicate (2 min apart) in the non-dominant arm using a validated semi-automatic oscillometer (Omron HEM-705CP, Hoofddorp, The Netherlands). For statistical analysis, the three measures’ average was noted and used.

### 2.6. Blood Collection and Analysis

After a 12 h overnight fast, venous blood and single spot urine samples were taken the following morning. Blood was drawn from the antecubital vein using a venous catheter and placed in the appropriate vacutainers. Fasting glucose, aspartate aminotransferase (AST), alanine aminotransferase (ALT), gamma-glutamyl transferase (GGT), high-density lipoprotein cholesterol (HDL-c), and triglyceride (TG) were among the normal laboratory parameters assessed on the Abbott ARCHITECT c16000 using commercial kits (Abbott Diagnostics, IL, USA).

Fatty liver index (FLI) allows the identification of NAFLD, and the prediction of future cases [28] and the Hepatic Steatosis Index (HSI) is a screening test for NAFLD [29]. Both were computed in the present study to determine the condition of fatty liver using non-invasive approaches. Cut-offs that showed NAFLD were FLI ≥ 60 and HSI ≥ 36. HSI = 30–36 were regarded as having an intermediate status.

### 2.7. Imaging

Using abdominal magnetic resonance imaging (MRI, Signa Explorer 1.5T, General Electric Healthcare, Chicago, IL, USA), liver fat was confirmed and measured as mean percentage (%). A mean intrahepatic fat content ≥ 6.4% was considered clinically significant [30]. 

Two groups were formed according to changes in intrahepatic fat content: participants that increased intrahepatic fat content (IIFC) and participants that reduced intrahepatic fat content (RIFC).

### 2.8. Statistics

Analyses were performed using the Statistical Package for Social Sciences (SPSS) version 27.0 (SPSS Inc., Chicago, IL, USA). Chi-square was used for the sociodemographic characteristics of the participants at baseline according to the two groups of changes in intrahepatic fat content. Continuous variables as means are represented as ±SD. Categorical data were expressed as count and percentage. Normality of data was assessed using the Kolmogorov–Smirnov test. A paired sample *t*-test was used to compare changes between COVID-19 before and after the lockdown for MetS and NAFLD parameters, and the consumption of food groups within each of the two intrahepatic fat content difference groups. To assess changes during the COVID-19 lockdown in MetS and NAFLD parameters, and consumption of food groups according to two intrahepatic fat content groups, a generalized linear model for repeated measures was used. All *p*-values were two-tailed with *p* < 0.05. 

## 3. Results

Table 1 shows sociodemographic characteristics from baseline participants according to changes in intrahepatic fat content. The obtained results showed that both considered populations (IIFC and RIFC) are not different from the point of view of their sociodemographic characteristics.

Table 2 shows changes in MetS parameters between pre- and post-lockdown. Analysing time*group differences, the IIFC group increased values of MetS parameters (body weight, WC, and fasting glucose) as a consequence of lockdown, whereas the RIFC group decreased the values of MetS parameters. Analysing changes within each group between pre- and post-lockdown, the IIFC group showed increases in body weight, body mass index, systolic and diastolic blood pressure, and fasting glucose, and the RIFC group just showed a significant decrease in waist circumference.

Table 3 shows changes in NAFLD parameters between pre- and post-lockdown. Lockdown negatively impacted NAFLD parameters, and time*group differences were observed in ALT, intrahepatic fat content by magnetic resonance imaging (IFC-MRI), and fatty liver index (FLI). Analysing changes in each group between pre- and post-lockdown, the IIFC group showed an increase in ALT, GGT, intrahepatic fat content by magnetic resonance imaging, fatty liver index, and hepatic steatosis index (HSI). The RIFC group and the RIFC group showed a significant decrease in ALT and IFC-NMR.

Oxidative stress was assessed (data not shown). SOD (superoxide dismutase) and CAT (catalase) enzymatic activities, as well as MDA (malondialdehyde) content, were assessed. In the present study, these did not register any significant change before and after the COVID-19 lockdown.

Table 4 shows the changes in macronutrients between pre and post lockdown. No differences time*group were observed. Analysing changes in each group between pre- and post-lockdown, fibre decreased in the IIFC group, trans-fats increased in both groups IIFC and RIFC, but no differences were registered.

Table 5 shows the changes in food consumption between pre- and post-lockdown. Lockdown modified consumption of several food groups; so, time*group differences were observed in the consumption of cheese, sweets and pastries, chocolates, and caffeinated coffee.

Analysing changes in each group between pre- and post-lockdown, the IIFC group decreased cheese, nuts, and caffeinated coffee consumption, but increased sweets and pastries consumption, whereas the RIFC group just decreased the consumption of cereals.

## 4. Discussion

In the current study, COVID-19 lockdown had a negative impact on MetS and NAFLD parameters, as well as on dietary aspects. By comparing pre- and post-lockdown periods, participants in the IIFC group showed a significant increase in body weight, systolic and diastolic blood pressure, fasting glycemia, ALT, GGT, intrahepatic fat content by NMR, fatty liver index, and hepatic steatosis index. Current findings are in line with a previous study in Italy which found that, after lockdown, participants worsened steatosis from mild to moderate or severe, and experienced an increase in body weight and an increase in fasting glycemia compared to the pre-lockdown period [31]. Another previous study carried out in participants living in European countries found that the high level of social isolation paved the way for people to live unhealthy lifestyles or have work as an exacerbator of existing metabolic problems [24]. In the current study, an increase in body weight was found, similar to other previous studies where weight gain occurred [31,32,33,34]. The first and most effective treatment for MetS and NAFLD is weight loss, achieved by diet and physical exercise [35]. Weight loss, especially if >5–10% from baseline, promotes improvement in hepatic steatosis, inflammation, and fibrosis [36]. In the current study, the mean weight gain was only 3 kg, and a worsening of hepatic steatosis occurred in the current population. Moreover, the presence of COVID-19 infection and NAFLD increased the risk of liver injury, as well as the presence of NAFLD, which could affect the outcome of COVID-19 [32,37]. NAFLD was a potential risk factor for SARS-CoV-2 infection and severe COVID-19, independent of metabolic syndrome [38].

Furthermore, participants who improved their liver status—participants in the RIFC group—ameliorated their ALT values, waist circumference, and intrahepatic fat content, assessed by magnetic resonance imaging post-lockdown. Current findings were aligned with another previous study, where participants who maintained adequate compliance to healthy lifestyles in the months before and during the lockdown showed an evident decrease in ultrasonographic fatty liver score [32]. This could be an example of maintaining healthy habits during lockdown; therefore, emphasising the importance of intensive educational campaigns during lockdown pandemic could make a difference.

In the present study, no differences in oxidative stress were found before and after confinement in any group. NAFLD is related to increased oxidative stress, even in early stages of the disease [39]. On the other hand, oxidative stress is a risk factor for COVID-19 infection [40]. However, some authors have shown that oxidative stress was reduced due to confinement, as exposure to urban pollutants was reduced [41]. Therefore, the lack of difference in changes between both groups could be explained by the decrease in external oxidants, such as those related to pollution.

In the current study, several macronutrients and food groups were consumed similarly during lockdown and resembled those of a previous study [42]. Conversely, during lockdown, people increased their consumption of processed “comfort foods”, such as chocolate, chips, and snacks [21,22]. In the current analysed population, it was observed that after lockdown, in the IIFC group (increased intrahepatic fat content), there was a significantly lower consumption of fibre, cheese, nuts and coffee. In contrast, participants in this group exhibited a higher consumption of sweets and pastries, as well as in trans fats. Interestingly, participants in the RIFC group (reduced intrahepatic fat content) exhibited a higher consumption of chocolates, where dark chocolate (70% and more) was the one recommended to the participants and the one they reported to consume.

To reduce NAFLD, lifestyle changes including maintaining a nutritious diet and engaging in frequent physical activity are essential [43]. It is crucial to remove any dietary components, such as fructose, saturated fatty acids, carbohydrates with a high glycaemic index, and foods high in salt, that can contribute to NAFLD [44]. Sweets and pastries fit into these types of not recommended food. Contrary to this, it has been observed that some foods play a beneficial role. In the literature, fibre, nuts, skimmed cheese, coffee, and dark chocolate are described to have potential benefits for NAFLD, but studies are inconsistent. Regarding fibre, a previous study demonstrated that increasing fibre intake (soluble and insoluble) from 19 to 29 g/d decreased liver enzymatic activity, and improved hepatic steatosis in NAFLD patients, perhaps through changing intestinal permeability [45]. In our study, participants in the IIFC group lowered their consumption to 28.8 g/d ± 12.2, which is a good amount of fibre, but due to the possible benefits of improving hepatic steatosis, it was associated with the enhancement of diverse metabolic diseases, including NAFLD [46]. However, this last study was performed in rodents, so further research should be carried out in humans to confirm these results. In any case, it would be important to maintain good amounts of fibre, especially during lockdowns. Moreover, the relationship between dietary fibre intake and NAFLD has also been reported in several previous basic and clinical studies in humans [47,48,49,50,51].

Concerning cheese, it is a rich source of so-called bioactive peptides [52] and probiotics. It seems that cheese could play an important role in the amelioration of NAFLD. A previous study found out that intakes of probiotic dairy products decrease NAFLD risk [53]. A previous study in rats concluded that cheese improved lipid metabolism, most likely because of a decrease in the accumulation of liver fat [54]; further studies in humans should be carried out to confirm these results in humans. Some lactic acid bacteria, including probiotics, appear to be able to bind to bile acids and boost their excretion, which reduces bile acid recycling in the enterohepatic circulation system and prevents micelle production in the gut [55]. In addition, a previous study in humans concluded that a higher dairy protein intake was inversely and significantly related to the likelihood of developing NAFLD [56].

Nut consumption has positive effects on health because they contain essential fatty acids such as monounsaturated fatty acids and polyunsaturated fatty acids (PUFA), as well as fat-soluble bioactive substances such as tocopherols, tocotrienols, phytosterols, sphingolipids, carotenoids, and chlorophyll [57,58]. Nevertheless, a previous study concluded that nut consumption was not associated with NAFLD risk in the overall sample [56]. According to another study, there is no relationship between eating nuts and the risk of developing NAFLD [59]. Contrary to the mentioned results, nuts lowered inflammation, improved lipid profiles, reduced insulin resistance, and improved glycaemic management, and may help to decrease the incidence of NAFLD [60,61,62,63].

Caffeine use was described as a protector against NAFLD [64,65,66,67]. The literature suggested that regular coffee has beneficial effects on the liver by lowering liver enzymes (GGT, ALT) and it was associated with reducing hepatic fibrosis in patients with NAFLD [64,65,68]. However, the protective role of coffee in NAFLD is still controversial due to designs, methods, amount of caffeine or type of coffee used, and intakes need to be quantified to generate an effective advantage.

Consuming chocolate, particularly dark chocolate, is linked to reduced lipid peroxidation, due to several polyphenolic compounds including epicatechin, a known natural antioxidant, and thereby improving cardiovascular risk [69,70]. Nevertheless, results concerning dark chocolate and NAFLD are very conflicting. A previous study in Baja California used 84 young participants who consumed either 2 g of milk chocolate or 2 g of dark chocolate with 70% cocoa every day for six months, and the study showed that the flavonoids in dark chocolate dramatically reduced DNA damage; increased cell nucleus integrity; improved TG, total and LDL-cholesterol levels; and reduced waist circumference [71]. In the current population, participants in the RIFC group reported the consumption of more dark chocolate ≥70% post-lockdown. However, previous studies showed that daily dark chocolate consumption did not result in any weight change [72]. Therefore, more research is needed to evaluate the effectiveness of dark chocolate before making recommendations. Whether the consumption of the above foods could be considered a preventative measure against NAFLD, further investigations should be directed at understanding the potential beneficial components of these foods and NAFLD; recommendations addressed to these patients could help reduce stress especially in lockdowns.

## 5. Strengths and Limitations

The main strength of this study is the use of magnetic resonance imaging to obtain liver scans, which is the most sensitive and precise non-invasive technique for measuring liver fat amounts [73]. The literature has drawn attention to the harmful consequences lockdown has on health; nevertheless, this study helps to highlight the effects on liver and metabolic syndrome parameters, and dietary patterns on participants with NAFLD and MetS, which are vulnerable to nutritional changes during pandemics. On the other side, the sample size was a major limitation. A larger sample size might produce more reliable results. Even though the fatty liver index, hepatic steatosis index, and Food Frequency Questionnaire have been validated, they still have limits due to their subjectivity. Other limiting factors include the participants’ various motivations, mental health, and physical health. Oxidative stress and inflammatory status have been assessed through general parameters, rather than liver-specific parameters, which could provide a more accurate status of liver inflammation and oxidative stress. Lastly, people were between 40 and 60 years old, which makes it difficult to extrapolate results to the general population; when extrapolating the findings to other regions of Spain or Europe, researchers and interventionists need to proceed with caution.

## 6. Conclusions

The COVID-19 lockdown led to negative changes in the liver of patients with NAFLD and MetS, with weight gain and increases in glycemia, ALT and intrahepatic fat content post-lockdown. Participants with worsened liver status exhibited low consumption of fibre, cheese, nuts and coffee, and high consumption of sweets and pastries. Participants who improved liver status ameliorated ALT values, waist circumference, and intrahepatic fat content, assessed by magnetic resonance imaging post-lockdown.

Maintaining good lifestyle practices is crucial, especially for populations with NAFLD and MetS, to reduce the unhealthy lifestyle patterns displayed during lockdown. Educational campaigns during lockdown are essential to minimise the negative effects of the pandemic on dietary habits.

## Figures and Tables

**Table 1:** Baseline sociodemographic characteristics of participants.

	IIFC n = 39	RIFC n = 20	*p*-Value *
Females (n; %)	15 (25.9)	6 (10.3)	0.362
Females on menopause (n; %)	6 (10.3)	4 (6.9)	0.385
Age (y) (mean ± SD)	51.4 ± 6.3	54.7 ± 6.7	0.072
Marital Status			0.842
Single (n; %)	3 (5.2)	1 (1.7)	
Married/domestic partnership (n; %)	26 (44.8)	16 (27.6)	
Divorced/separated/widowed (n; %)	10 (17.2)	3 (5.2)	
Employment			0.254
Working (n; %)	32 (55.2)	15 (25.9)	
Unemployed/retired/housewife (n; %)	7 (12.1)	5 (8.5)	
Education Level			0.637
University/post-university (n; %)	3 (5.2)	1 (1.7)	
Secondary education (n; %)	9 (15.5)	7 (12.1)	
Primary education (n; %)	15 (25.8)	10 (17.2)	
None (n; %)	12 (20.7)	2 (3.4)	
Currently smoking (n; %)	7 (12.1)	0 (0.0)	0.190
Regular Physical Activity			0.253
None (n; %)	19 (32.8)	7 (12.1)	
Light (n; %)	15 (25.9)	9 (15.5)	
Moderate (n; %)	4 (6.9)	4 (6.9)	
Vigorous (n; %)	1 (1.7)	0 (0.0)	
T2DM (n; %)	10 (17.2)	8 (13.8)	0.381
High BP (n; %)	15 (30.6)	10 (20.4)	0.858

Abbreviations: BP: blood pressure; IIFC: increased intrahepatic fat content; RIFC: reduced intrahepatic fat content; SD: standard deviation; T2DM: type 2 diabetes mellitus. Data are expressed as count (%), unless otherwise specified. * differences IIFC vs. RIFC by chi-square.

**Table 2:** Changes in metabolic syndrome parameters between pre- and post-lockdown.

		IIFC Mean (SD)	RIFC Mean (SD)	t*g†
BMI (kg/m^2^)	Pre-lockdown	31.7 ± 3.3	33.0 ± 3.6	0.241
	Post-lockdown	32.7 ± 3.5	44.9 ± 56.0	
	Δ	+1.1 ± 1.9 *	+11.9 ± 55.6	
Body weight (kg)	Pre-lockdown	89.0 ± 11.8	91.9 ± 10.1	<0.001
	Post-lockdown	91.8 ± 12.0	90.5 ± 12.2	
	Δ	+2.8 ± 2.9 *	−1.4 ± 4.2	
WC (cm)	Pre-lockdown	106.9 ± 8.1	111.4 ± 9.3	0.002
	Post-lockdown	108.1 ± 7.9	108.6 ± 9.0	
	Δ	+1.2 ± 4.4	−2.9 ± 4.4 *	
SBP (mmHg)	Pre-lockdown	131.1 ± 12.9	131.3 ± 17.4	0.129
	Post-lockdown	136.3 ± 18.4	130.5 ± 18.0	
	Δ	+5.2 ± 13.3 *	−0.8 ± 13.0	
DBP (mmHg)	Pre-lockdown	80.4 ± 7.4	78.7 ± 7.6	0.215
	Post-lockdown	85.9 ± 11.5	81.1 ± 10.8	
	Δ	+5.5 ± 8.0 *	+2.4 ± 9.1	
Fasting glucose (mg/dL)	Pre-lockdown	100.5 ± 14.0	118.4 ± 42.1	0.019
	Post-lockdown	107.3 ± 19.3	112.6 ± 41.5	
	Δ	+6.8 ± 13.5 *	−5.8 ± 25.3	
HDL-c (mg/dL)	Pre-lockdown	47.0 ± 10.9	42.8 ± 6.5	0.605
	Post-lockdown	45.3 ± 9.6	41.8 ± 6.4	
	Δ	−1.7 ± 5.8	−1.0 ± 4.3	
TG (mg/dL)	Pre-lockdown	166.9 ± 121.3	154.9 ± 80.7	0.410
	Post-lockdown	216.5 ± 266.9	163.8 ± 63.8	
	Δ	+49.6 ± 215.7	+8.9 ± 77.4	

Abbreviations: BMI: body mass index; Δ: delta between pre- and post-lockdown; DBP: diastolic blood pressure; HDL-c: high density lipoprotein-cholesterol; IIFC: increased intrahepatic fat content; RIFC: reduced intrahepatic fat content; SBP: systolic blood pressure; SD: standard deviation; TG: triglycerides. t*g†: difference Δ-IIFC vs. Δ-RIFC time*group by one-way repeated measures ANCOVA. * differences pre-lockdown vs. post-lockdown by Student’s *t*-test.

**Table 3:** Changes in NAFLD parameters between pre- and post-lockdown.

		IIFC Mean (SD)	RIFC Mean (SD)	t*g†
AST (U/L)	Pre-lockdown	21.7 ± 6.7	25.9 ± 7.9	0.077
	Post-lockdown	27.9 ± 19.4	24.4 ± 7.2	
	Δ	+6.2 ± 16.6	−1.6 ± 5.3	
ALT (U/L)	Pre-lockdown	25.0 ± 9.8	32.0 ± 14.0	0.020
	Post-lockdown	36.1 ± 30.6	29.1 ± 14.0	
	Δ	+11.1 ± 25.4 *	−2.9 ± 11.2 *	
GGT (U/L)	Pre-lockdown	36.1 ± 22.6	38.4 ± 24.3	0.196
	Post-lockdown	50.8 ± 52.3	41.0 ± 39.3	
	Δ	+14.7 ± 38.6 *	+2.6 ± 22.0	
IFC-MRI (%)	Pre-lockdown	10.0 ± 7.7	14.4 ± 8.7	<0.001
	Post-lockdown	14.0 ± 9.6	11.3 ± 7.2	
	Δ	+4.0 ± 3.4 *	−3.0 ± 3.3 *	
FLI (U)	Pre-lockdown	77.2 ± 15.7	83.1 ± 12.1	0.013
	Post-lockdown	82.9 ± 14.8	82.4 ± 17.2	
	Δ	+5.7 ± 7.7 *	−0.7 ± 10.8	
HIS (U)	Pre-lockdown	42.3 ± 4.7	43.8 ± 5.8	0.255
	Post-lockdown	44.4 ± 5.0	59.2 ± 63.2	
	Δ	+2.0 ± 2.6 *	+15.4 ± 62.9	

Abbreviations: ALT: alanine aminotransferase; AST: aspartate aminotransferase; Δ: delta between pre and post lockdown; FLI: fatty liver index; GGT: gamma-glutamyl transferase; HSI: hepatic steatosis index; IFC-MRI: intrahepatic fat content by magnetic resonance imaging; IIFC: increased intrahepatic fat content; NAFLD: non-alcoholic fatty liver disease; RIFC: reduced intrahepatic fat content; SD: standard deviation; U: units. t*g†: difference Δ-IIFC vs. Δ-RIFC time*group by one-way repeated measures ANCOVA. * differences pre-lockdown vs. post-lockdown by Student’s *t*-test.

**Table 4:** Changes in macronutrients at pre- and post-lockdown.

		IIFC Mean (SD)	RIFC Mean (SD)	t*g†
Carbohydrates (g/d) per 1000 kcal	Pre-lockdown	210.0 ± 81.0	214.0 ± 62.2	0.943
	Post-lockdown	189.1 ± 65.0	194.2 ± 62.9	
	Δ	−20.9 ± 55.6	−19.8 ± 49.6	
Fibre (g/d) per 1000 kcal	Pre-lockdown	31.8 ± 12.5	31.9 ± 12.3	0.343
	Post-lockdown	28.8 ± 12.2	31.1 ± 10.8	
	Δ	−3.0 ± 7.1 *	−0.8 ± 8.5	
Protein (g/d) per 1000 kcal	Pre-lockdown	98.2 ± 26.8	91.1 ± 27.1	0.508
	Post-lockdown	90.5 ± 28.2	88.3 ± 22.4	
	Δ	−7.6 ± 26.8	−2.8 ± 20.3	
Lipids (g/d) per 1000 kcal	Pre-lockdown	82.9 ± 23.9	89.8 ± 27.6	0.385
	Post-lockdown	92.5 ± 32.2	92.0 ± 27.5	
	Δ	+9.6 ± 32.6	+2.2 ± 21.3	
Trans fats (g/d) per 1000 kcal	Pre-lockdown	5.6 ± 6.5	7.9 ± 6.8	0.979
	Post-lockdown	9.4 ± 4.3	11.7 ± 5.6	
	Δ	+3.8 ± 7.1 *	+3.8 ± 7.6 *	

Abbreviations: IIFC: increased intrahepatic fat content; RIFC: reduced intrahepatic fat content; SD: standard deviation; Δ: delta between pre and post lockdown; Adherence to MedDiet: adherence to Mediterranean diet. t*g†: difference Δ-IIFC vs. Δ-RIFC time*group by one-way repeated measures ANCOVA. * differences pre-lockdown vs. post-lockdown by Student’s *t*-test.

**Table 5:** Changes in food groups between pre and post lockdown.

		IIFC Mean (SD)	RIFC Mean (SD)	t*g†
Fruits + vegetables (g/d) per 1000kcal	Pre-lockdown	715.0 ± 304.9	693.2 ± 258.8	0.805
	Post-lockdown	666.6 ± 321.9	660.4 ± 212.6	
	Δ	−48.5 ± 227.3	−32.8 ± 192.0	
Cereals (g/d) per 1000 kcal	Pre-lockdown	125.9 ± 67.6	144.1 ± 67.8	0.530
	Post-lockdown	113.4 ± 57.5	119.9 ± 67.0	
	Δ	−12.6 ± 70.8	−24.2 ± 46.0 *	
Legumes (g/d) per 1000 kcal	Pre-lockdown	31.2 ± 17.8	30.2 ± 24.3	0.802
	Post-lockdown	34.4 ± 25.7	31.8 ± 27.0	
	Δ	+3.1 ± 21.8	+1.6 ± 19.1	
Milk and yogurt (mL/d) per 1000 kcal	Pre-lockdown	264.2 ± 189.4	232.4 ± 141.4	0.299
	Post-lockdown	239.6 ± 158.2	262.8 ± 185.8	
	Δ	−24.5 ± 183.9	+30.3 ± 165.0	
Cheese (g/d) per 1000 kcal	Pre-lockdown	9.6 ± 9.7	8.6 ± 10.2	0.011
	Post-lockdown	6.0 ± 7.6	10.9 ± 10.4	
	Δ	−3.6 ± 8.4 *	+2.3 ± 5.9	
Meats and meat products (g/d) per 1000 kcal	Pre-lockdown	126.1 ± 65.2	130.6 ± 69.8	0.615
	Post-lockdown	111.2 ± 69.0	126.9 ± 48.3	
	Δ	−14.9 ± 84.6	−3.7 ± 57.0	
Fish (g/d) per 1000 kcal	Pre-lockdown	147.2 ± 76.5	101.5 ± 60.1	0.639
	Post-lockdown	146.3 ± 82.9	94.0 ± 62.9	
	Δ	−0.9 ± 40.4	−7.5 ± 56.9	
Nuts (g/d) per 1000 kcal	Pre-lockdown	25.2 ± 29.3	27.3 ± 23.6	0.233
	Post-lockdown	14.6 ± 16.4	26.3 ± 24.8	
	Δ	−10.5 ± 26.3 *	-1.0 ± 27.2	
Cooking oils (mg/d) per 1000 kcal	Pre-lockdown	32.4 ± 15.1	35.3 ± 16.4	0.934
	Post-lockdown	33.6 ± 13.2	35.6 ± 16.0	
	Δ	+1.2 ± 13.8	+0.3 ± 16.6	
Sweets and pastries (g/d) per 1000 kcal	Pre-lockdown	7.6 ± 8.0	7.5 ± 11.8	0.040
	Post-lockdown	17.7 ± 35.7	14.4 ± 26.1	
	Δ	+10.1 ± 32.1 *	+6.9 ± 23.8	
Chocolates (g/d) per 1000 kcal	Pre-lockdown	3.4 ± 6.8	2.4 ± 5.3	0.047
	Post-lockdown	5.0 ± 6.1	12.6 ± 34.6	
	Δ	+1.6 ± 8.2	+10.2 ± 29.6	
Soft drinks (mL/d) per 1000 kcal	Pre-lockdown	94.3 ± 126.8	86.1 ± 130.1	0.645
	Post-lockdown	129.1 ± 181.6	99.3 ± 158.1	
	Δ	+34.8 ± 166.7	+13.3 ± 141.3	
Caffeinated coffee (mL/d) per 1000 kcal	Pre-lockdown	67.1 ± 73.3	36.5 ± 50.4	0.006
	Post-lockdown	40.5 ± 48.8	41.9 ± 54.1	
	Δ	−26.6 ± 46.5 *	+5.5 ± 18.1	

Abbreviations: IIFC: increased intrahepatic fat content; RIFC: reduced intrahepatic fat content; SD: standard deviation; Δ: delta between pre- and post-lockdown; Adherence to MedDiet: adherence to Mediterranean diet. t*g†: difference Δ-IIFC vs. Δ-RIFC time*group by one-way repeated measures ANCOVA. * differences pre-lockdown vs. post-lockdown by Student’s *t*-test.

## Data Availability

There are restrictions on the availability of data for this trial, due to the signed consent agreements around data sharing, which only allow access to external researchers for studies following the project’s purposes. Requestors wishing to access the trial data used in this study can make a request to pep.tur@uib.es.

## References

[1] Benedict M., Zhang X. (2017). Non-alcoholic fatty liver disease: An expanded review. J. Hepatol..

[2] Mitra S., De A., Chowdhury A. (2020). Epidemiology of non-alcoholic and alcoholic fatty liver diseases. Transl. Gastroenterol. Hepatol..

[3] Allen A.M., Therneau T.M., Larson J.J., Coward A., Somers V.K., Kamath P.S. (2018). Nonalcoholic Fatty Liver Disease Incidence and Impact on Metabolic Burden and Death: A 20 Year-Community Study. Hepatology.

[4] Le M.H., Yeo Y.H., Li X., Li J., Zou B., Wu Y., Ye Q., Huang D.Q., Zhao C., Zhang J. (2021). 2019 Global NAFLD Prevalence: A Systematic Review and Meta-analysis. Clin. Gastroenterol. Hepatol..

[5] Sayiner M., Koenig A., Henry L., Younossi Z.M. (2016). Epidemiology of Nonalcoholic Fatty Liver Disease and Nonalcoholic Steatohepatitis in the United States and the Rest of the World. Clin. Liver Dis..

[6] Kanwar P., Kowdley K.V. (2016). The Metabolic Syndrome and Its Influence on Nonalcoholic Steatohepatitis. Clin. Liver Dis..

[7] Vanni E., Bugianesi E., Kotronen A., De Minicis S., Yki-Järvinen H., Svegliati-Baroni G. (2010). From the metabolic syndrome to NAFLD or vice versa?. Dig. Liver Dis..

[8] Alberti K.G.M.M., Eckel R.H., Grundy S.M., Zimmet P.Z., Cleeman J.I., Donato K.A., Fruchart J.C., James W.P.T., Loria C.M., Smith S.C. (2009). Harmonizing the metabolic syndrome: A joint interim statement of the International Diabetes Federation Task Force on Epidemiology and Prevention; National Heart, Lung, and Blood Institute; American Heart Association; World Heart Federation; International Atherosclerosis Society; and International Association for the Study of Obesity. Circulation.

[9] Dumas M.E., Kinross J., Nicholson J.K. (2014). Metabolic phenotyping and systems biology approaches to understanding metabolic syndrome and fatty liver disease. Gastroenterology.

[10] Sachdeva S., Khandait H., Kopel J., Aloysius M.M., Desai R., Goyal H. (2020). NAFLD and COVID-19: A Pooled Analysis. Gastroenterology.

[11] Bao Y., Sun Y., Meng S., Shi J., Lu L. (2020). 2019-nCoV epidemic: Address mental health care to empower society. Lancet.

[12] Abbas A.M., Kamel M.M. (2020). Dietary habits in adults during quarantine in the context of COVID-19 pandemic. Obes. Med..

[13] Pellegrini M., Ponzo V., Rosato R., Scumaci E., Goitre I., Benso A., Belcastro S., Crespi C., De Michieli F., Ghigo E. (2020). Changes in weight and nutritional habits in adults with obesity during the “lockdown” period caused by the COVID-19 virus emergency. Nutrients.

[14] Galanakis C.M. (2020). The food systems in the era of the coronavirus (COVID-19) pandemic crisis. Foods.

[15] Pérez-Rodrigo C., Gianzo-Citores M., Hervás-Bárbara G., Ruis-Litago F., Casís-Sáenz L., Arija V., López-Sobaler A.M., Martínez de Victoria E., Ortega R.M., Partearroyo T. (2021). Patterns of Change in Dietary Habits and Physical Activity during Lockdown in Spain Due to the COVID-19 Pandemic. Nutrients.

[16] Sánchez-Sánchez E., Ramirez-Vargas G., Avellaneda-López Y., Orellana-Pecino J.I., García-Marín E., Díaz-Jiménez J. (2020). Eating Habits and Physical Activity of the Spanish Population during the COVID-19 Pandemic Period. Nutrients.

[17] Mattioli A.V., Sciomer S., Cocchi C., Maffei S., Gallina S. (2020). Quarantine during COVID-19 outbreak changes in diet and physical activity increase the risk of cardiovascular disease. Nutr. Metab. Cardiovasc. Dis..

[18] Casas R., Raicó-Quintana B., Ruiz-León A.M., Castro-Barquero S., Bertomeu I., González-Juste J., Campolier M., Estruch R. (2022). Changes in Spanish lifestyle and dietary habits during the COVID-19 lockdown. Eur. J. Nutr..

[19] Mascherini G., Catelan D., Pellegrini-Giampietro D.E., Petri C., Scaletti C., Gulisano M. (2021). Changes in physical activity levels, eating habits and psychological well-being during the Italian COVID-19 pandemic lockdown: Impact of socio-demographic factors on the Florentine academic population. PLoS ONE.

[20] Lombardo M., Guseva E., Perrone M.A., Müller A., Rizzo G., Storz M.A. (2021). Changes in Eating Habits and Physical Activity after COVID-19 Pandemic Lockdowns in Italy. Nutrients.

[21] Bracale R., Vaccaro C.M. (2020). Changes in food choice following restrictive measures due to COVID-19. Nutr. Metab. Cardiovasc. Dis..

[22] Scarmozzino F., Visioli F. (2020). COVID-19 and the subsequent lockdown modified dietary habits of almost half the population in an Italian sample. Foods.

[23] Di Renzo L., Gualtieri P., Cinelli G., Bigioni G., Soldati L., Attinà A., Bianco F.F., Caparello G., Camodeca V., Carrano E. (2020). Psychological aspects and eating habits during COVID-19 home confinement: Results of EHLC-COVID-19 Italian online survey. Nutrients.

[24] (2020). Prevention and Reversion of NAFLD in Obese Patients with Metabolic Syndrome by Mediterranean Diet and Physical Activity (FLIPAN). https://clinicaltrials.gov/ct2/show/NCT04442620.

[25] Consensus International Diabetic Federation (IDF) (2006). Consensus Statement—The IDF Consensus Worldwide Definition of the Metabolic Syndrome. https://www.idf.org/e-library/consensus-statements/60-idfconsensus-worldwidedefinitionof-the-metabolic-syndrome.html.

[26] Willett W.C., Sampson L., Stampfer M.J., Rosner B., Bain C., Witschi J., Hennekens C.H., Speizer F.E. (1985). Reproducibility and validity of a semiquantitative food frequency questionnaire. Am. J. Epidemiol..

[27] Moreiras O., Carbajal A., Cabrera L., Cuadrado C. (2018). Food Composition Tables (Spanish).

[28] Bedogni G., Bellentani S., Miglioli L., Masutti F., Passalacqua M., Castiglione A., Tiribelli C. (2006). The Fatty Liver Index: A simple and accurate predictor of hepatic steatosis in the general population. BMC Gastroenterol..

[29] Lee J.H., Kim D., Kim H.J., Lee C.H., Yang J.I., Kim W., Kim Y.J., Yoon J.H., Cho S.H., Sung M.W. (2010). Hepatic steatosis index: A simple screening tool reflecting nonalcoholic fatty liver disease. Dig. Liver Dis..

[30] Tang A., Tan J., Sun M., Hamilton G., Bydder M., Wolfson T., Gamst A.C., Middleton M., Brunt E.M., Loomba R. (2013). Nonalcoholic fatty liver disease: MR imaging of liver proton density fat fraction to assess hepatic steatosis. Radiology.

[31] Cinque F., Cespiati A., Lombardi R., Costantino A., Maffi G., Alletto F., Colavolpe L., Francione P., Oberti G., Fatta E. (2022). Interaction between Lifestyle Changes and PNPLA3 Genotype in NAFLD Patients during the COVID-19 Lockdown. Nutrients.

[32] Shanmugam H., Di Ciaula A., Di Palo D.M., Molina-Molina E., Garruti G., Faienza M.F., van Erpecum K., Portincasa P. (2021). Multiplying effects of COVID-19 lockdown on metabolic risk and fatty liver. Eur. J. Clin. Investig..

[33] Khan M.A., Menon P., Govender R., Samra A.M.A., Allaham K.K., Nauman J., Östlundh L., Mustafa H., Smith J.E.M., AlKaabi J.M. (2022). Systematic review of the effects of pandemic confinements on body weight and their determinants. Br. J. Nutr..

[34] Bennett G., Young E., Butler I., Coe S. (2021). The Impact of Lockdown During the COVID-19 Outbreak on Dietary Habits in Various Population Groups: A Scoping Review. Front. Nutr..

[35] European Association for the Study of the Liver (EASL), European Association for the Study of Diabetes (EASD), European Association for the Study of Obesity (EASO) (2016). EASL-EASD-EASO Clinical Practice Guidelines for the management of non-alcoholic fatty liver disease. Diabetologia.

[36] Vilar-Gomez E., Martinez-Perez Y., Calzadilla-Bertot L., Torres-Gonzalez A., Gra-Oramas B., Gonzalez-Fabian L., Friedman S.L., Diago M., Romero-Gomez M. (2015). Weight Loss Through Lifestyle Modification Significantly Reduces Features of Nonalcoholic Steatohepatitis. Gastroenterology.

[37] Ji D., Qin E., Xu J., Zhang D., Cheng G., Wang Y., Lau G. (2020). Non-alcoholic fatty liver diseases in patients with COVID-19: A retrospective study. J. Hepatol..

[38] Ahmed M., Ahmed M.H. (2021). Nonalcoholic fatty liver disease and COVID-19: An epidemic that begets pandemic. World. J. Clin. Cases..

[39] Smirne C., Croce E., Di Benedetto D., Cantaluppi V., Comi C., Sainaghi P.P., Minisini R., Grossini E., Pirisi M. (2022). Oxidative Stress in Non-Alcoholic Fatty Liver Disease. Livers.

[40] Galli F., Marcantonini G., Giustarini D., Albertini M.C., Migni A., Zatini L., Gioiello A., Rossi R., Bartolini D. (2022). How Aging and Oxidative Stress Influence the Cytopathic and Inflammatory Effects of SARS-CoV-2 Infection: The Role of Cellular Glutathione and Cysteine Metabolism. Antioxidants.

[41] Buonaurio F., Borra F., Pigini D., Paci E., Spagnoli M., Astolfi M.L., Giampaoli O., Sciubba F., Miccheli A., Canepari S. (2022). Biomonitoring of Exposure to Urban Pollutants and Oxidative Stress during the COVID-19 Lockdown in Rome Residents. Toxics.

[42] Janssen M., Chang B.P.I., Hristov H., Pravst I., Profeta A., Millard J. (2021). Changes in Food Consumption During the COVID-19 Pandemic: Analysis of Consumer Survey Data from the First Lockdown Period in Denmark, Germany, and Slovenia. Front. Nutr..

[43] Michel M., Schattenberg J.M. (2020). Effectiveness of lifestyle interventions in NAFLD (nonalcoholic fatty liver disease)-how are clinical trials affected?. Expert. Opin. Investig. Drugs..

[44] Abenavoli L., Procopio A.C., Medić-Stojanoska M., Luzza F. (2020). Non-alcoholic fatty liver disease and primary care physicians. Minerva. Gastroenterol. Dietol..

[45] Krawczyk M., Maciejewska D., Ryterska K., Czerwińka-Rogowska M., Jamioł-Milc D., Skonieczna-Żydecka K., Milkiewicz P., Raszeja-Wyszomirska J., Stachowska E. (2018). Gut Permeability Might be Improved by Dietary Fiber in Individuals with Nonalcoholic Fatty Liver Disease (NAFLD) Undergoing Weight Reduction. Nutrients.

[46] Parnell J.A., Raman M., Rioux K.P., Reimer R.A. (2011). The potential role of prebiotic fibre for treatment and management of non-alcoholic fatty liver disease and associated obesity and insulin resistance. Liver Int..

[47] Rietman A., Sluik D., Feskens E.J.M., Kok F.J., Mensink M. (2018). Associations between dietary factors and markers of NAFLD in a general Dutch adult population. Eur. J. Clin. Nutr..

[48] Xia Y., Zhang S., Zhang Q., Liu L., Meng G., Wu H., Bao X., Gu Y., Sun S., Wang X. (2020). Insoluble dietary fibre intake is associated with lower prevalence of newly-diagnosed non-alcoholic fatty liver disease in Chinese men: A large population-based cross-sectional study. Nutr. Metab..

[49] Zolfaghari H., Askari G., Siassi F., Feizi A., Sotoudeh G. (2016). Intake of nutrients, fiber, and sugar in patients with nonalcoholic fatty liver disease in comparison to healthy individuals. Int. J. Prev. Med..

[50] Zelber-Sagi S., Nitzan-Kaluski D., Goldsmith R., Webb M., Blendis L., Halpern Z., Oren R. (2007). Long term nutritional intake and the risk for non-alcoholic fatty liver disease (NAFLD): A population based study. J. Hepatol..

[51] Yang Z., Wu J., Li X., Xie D., Wang Y., Yang T. (2019). Association between dietary iron intake and the prevalence of nonalcoholic fatty liver disease: A cross-sectional study. Medicine.

[52] Moller N.P., Scholz-Ahrens K.E., Roos N., Schrezenmeir J. (2008). Bioactive peptides and proteins from foods: Indication for health effects. Eur. J. Nutr..

[53] Koutnikova H., Genser B., Monteiro-Sepulveda M., Faurie J.M., Rizkalla S., Schrezenmeir J., Clément K. (2019). Impact of bacterial probiotics on obesity, diabetes and non-alcoholic fatty liver disease related variables: A systematic review and meta-analysis of randomised controlled trials. BMJ Open..

[54] Higurashi S., Ogawa A., Nara T.Y., Kato K., Kadooka Y. (2016). Cheese consumption prevents fat accumulation in the liver and improves serum lipid parameters in rats fed a high-fat diet. Dairy Sci. Technol..

[55] St-Onge M.P., Farnworth E.R., Jones P.J. (2000). Consumption of fermented and nonfermented dairy products: Effects on cholesterol concentrations and metabolism. Am. J. Clin. Nutr..

[56] Lee J.H., Lee H.S., Ahn S.B., Kwon Y.J. (2021). Dairy protein intake is inversely related to development of non-alcoholic fatty liver disease. Clin. Nutr..

[57] Alasalvar C., Bolling B.W. (2015). Review of nut phytochemicals, fat-soluble bioactives, antioxidant components and health effects. Br. J. Nutr..

[58] Alasalvar C., Salas-Salvadó J., Ros E. (2020). Bioactives and health benefits of nuts and dried fruits. Food Chem..

[59] Chen B.B., Han Y., Pan X., Yan J., Liu W., Li Y., Lin X., Xu S., Peng X.E. (2019). Association between nut intake and non-alcoholic fatty liver disease risk: A retrospective case-control study in a sample of Chinese Han adults. BMJ Open..

[60] Asbaghi O., Emamat H., Kelishadi M.R., Hekmatdoost A. (2020). The Association between Nuts Intake and Non-Alcoholic Fatty Liver Disease (NAFLD) Risk: A Case-Control Study. Clin. Nutr. Res..

[61] Grosso G., Yang J., Marventano S., Micek A., Galvano F., Kales S.N. (2015). Nut consumption on all-cause, cardiovascular, and cancer mortality risk: A systematic review and meta-analysis of epidemiologic studies. Am. J. Clin. Nutr..

[62] Tindall A.M., Johnston E.A., Kris-Etherton P.M., Petersen K.S. (2019). The effect of nuts on markers of glycemic control: A systematic review and meta-analysis of randomized controlled trials. Am. J. Clin. Nutr..

[63] Del Gobbo L.C., Falk M.C., Feldman R., Lewis K., Mozaffarian D. (2015). Effects of tree nuts on blood lipids, apolipoproteins, and blood pressure: Systematic review, meta-analysis, and dose-response of 61 controlled intervention trials. Am. J. Clin. Nutr..

[64] Molloy J.W., Calcagno C.J., Williams C.D., Jones F.J., Torres D.M., Harrison S.A. (2012). Association of coffee and caffeine consumption with fatty liver disease, nonalcoholic steatohepatitis, and degree of hepatic fibrosis. Hepatology.

[65] Birerdinc A., Stepanova M., Pawloski L., Younossi Z.M. (2012). Caffeine is protective in patients with non-alcoholic fatty liver disease. Aliment. Pharmacol. Ther..

[66] Catalano D., Martines G.F., Tonzuso A., Pirri C., Trovato F.M., Trovato G.M. (2010). Protective role of coffee in non-alcoholic fatty liver disease (NAFLD). Dig. Dis. Sci..

[67] Gutiérrez-Grobe Y., Chávez-Tapia N., Sánchez-Valle V., Gavilanes-Espinar J.G., Ponciano-Rodríguez G., Uribe M., Méndez-Sánchez N. (2012). High coffee intake is associated with lower grade nonalcoholic fatty liver disease: The role of peripheral antioxidant activity. Ann. Hepatol..

[68] Shen H., Rodriguez A.C., Shiani A., Lipka S., Shahzad G., Kumar A., Mustacchia P. (2016). Association between caffeine consumption and nonalcoholic fatty liver disease: A systemic review and meta-analysis. Therap. Adv. Gastroenterol..

[69] Kerimi A., Williamson G. (2015). The cardiovascular benefits of dark chocolate. Vascul. Pharmacol..

[70] McShea A., Ramiro-Puig E., Munro S.B., Casadesus G., Castell M., Smith M.A. (2008). Clinical benefit and preservation of flavonols in dark chocolate manufacturing. Nutr. Rev..

[71] Leyva-Soto A., Chavez-Santoscoy R.A., Lara-Jacobo L.R., Chavez-Santoscoy A.V., Gonzalez-Cobian L.N. (2018). Daily Consumption of Chocolate Rich in Flavonoids Decreases Cellular Genotoxicity and Improves Biochemical Parameters of Lipid and Glucose Metabolism. Molecules.

[72] Di Renzo L., Rizzo M., Sarlo F., Colica C., Iacopino L., Domino E., Sergi D., De Lorenzo A. (2013). Effects of dark chocolate in a population of Normal Weight Obese women: A pilot study. Eur. Rev. Med. Pharmacol. Sci..

[73] Lv S., Jiang S., Liu S., Dong Q., Xin Y., Xuan S. (2018). Non-invasive quantitative detection methods of liver fat content in non-alcoholic fatty liver disease. J. Clin. Transl. Hepatol..

